# A Mixed-Methods Study on Senior High School EFL Teacher Resilience in China

**DOI:** 10.3389/fpsyg.2022.865599

**Published:** 2022-04-27

**Authors:** Wenxiu Chu, Honggang Liu

**Affiliations:** School of Foreign Languages, Northeast Normal University, Changchun, China

**Keywords:** resilience, CD-RISC-EFL teacher survey, EFL teacher, senior high school, mixed methods

## Abstract

While teacher resilience has gained significant attention in recent years, empirical exploration of this issue is still insufficient, particularly with regard to English as a foreign language (EFL) teacher resilience in China. In this context, this study employed a mixed-methods design to investigate Chinese EFL teacher resilience. Specifically, the Connor-Davidson Resilience Scale (CD-RISC)-EFL Teacher Survey was distributed to 330 Chinese senior high school EFL teachers. Five volunteers in the survey sample participated in semi-structured interviews. The results revealed that resilience in Chinese senior high school EFL teachers was at a moderate to high level, and there was no significant difference in teacher resilience in relation to gender and years of teaching experience, but a small significant difference with teachers’ educational background. Possible reasons for interpreting EFL teacher resilience were provided from personal and contextual perspectives. Finally, the implications of these findings were discussed for developing teacher resilience.

## Introduction

Teacher resilience, as a newly emerged topic, has gained much attention in recent years (Gu and Day, 2013; Gu, 2018; Hiver, 2018; Mansfield et al., 2018; Fan et al., 2021; Zhang, 2021), especially within the booming perspective of positive psychology. This topic offers productive insight into what enables teachers to exercise their proactive agency to respond to challenges and survive and thrive in the educational context (Tait, 2008; Beltman et al., 2011; Li et al., 2019; Huang, 2021). Teaching is regarded as a highly demanding and labor-intensive job (Gu and Li, 2013; Zhang, 2021), particularly in the domain of language education. The waves of reform in language education have brought about new teaching challenges (Murray et al., 2020; Wen and Zhang, 2020). Language teachers experience pressures emanating from cultural differences, student misbehavior, and disharmonious interpersonal relationships, which contribute to their negative emotions and unstable working status (Talbot and Mercer, 2018; MacIntyre et al., 2019; Gkonou et al., 2020). Thus, an urgent need exists for cultivating resilient teachers who are able to effectively overcome teaching stress and maintain equilibrium in educational settings (Brunetti, 2006; Tait, 2008; Gu and Day, 2013; Gu, 2018). Furthermore, unpacking teachers’ capacity for resilience is conducive to interpreting their beliefs and agency, professional identity, and motivation to teach (Fenton-O’Creevy et al., 2015; Flores, 2018; Lohbeck, 2018; Huang, 2021), and to enhancing their teaching quality and efficiency (Gu and Day, 2013; Li et al., 2019). Further exploration of language teacher resilience also helps enrich studies on language teacher psychology (Mercer and Kostoulas, 2018). However, to date, research on teacher resilience is at its initial stage (Gu and Li, 2013; Gu, 2018; Wosnitza and Peixoto, 2018), with less attention paid to language teacher resilience (Kostoulas and Lämmerer, 2018; Fan et al., 2021).

Empirical research on language teacher resilience has primarily adopted a qualitative research design to explore the features of resilient teachers, factors influencing teacher resilience, and resilience strategies. Only a few studies endeavored to use quantitative (e.g., Ergün and Dewaele, 2021; Liu and Chu, 2022) or mixed research methods (Kostoulas and Lämmerer, 2020) to uncover language teacher resilience, such as its levels, and differences based on demographic variables, such as gender, years of teaching experience, and educational background. Meanwhile, these demographic factors are considered in teacher psychology research, such as in studies concerning motivation (Dewaele, 2020), emotional labor (Acheson and Nelson, 2020), and wellbeing (Kidger et al., 2016). For example, years of teaching experience become a necessary criterion for selecting excellent EFL teachers (Chu et al., 2021). However, a specific analysis of the role of sociobiographical variables in affecting teacher resilience is still lacking, and there is a need to deeply examine and interpret their relationships. To the best of our knowledge, very few studies to date have empirically investigated teacher resilience in the Chinese EFL teaching context (Fan et al., 2021; Liu and Chu, 2022). To expand this body of literature, the present study aims to utilize a mixed-methods design to explore EFL teacher resilience in China.

## Studies on Teacher Resilience: A Brief Review

### Defining Teacher Resilience

Although resilience has long been researched, especially in the field of child psychology, there is a lack of consensus on the definition of resilience. Precisely, resilience has been viewed as the personal quality or capacity that promotes individuals to survive, adjust, and flourish in spite of adversities (Rutter, 1987; Connor and Davidson, 2003; Yu and Zhang, 2007), while some scholars have argued that resilience is a dynamic and complex process or individuals’ behavioral outcomes of recovering and thriving in the face of stressful events (Masten et al., 1990; Luthar and Cicchetti, 2000). Resilience has also been defined as “a mode of interacting with events in the environment that is activated and nurtured in times of stress” (Tait, 2008, p. 58). However, overall, two core components encapsulate the concept of resilience: the presence of risks and positive adaptation or adjustment to these risks (Luthar and Cicchetti, 2000; Leroux, 2018).

Inspired by previous research on child resilience, teacher resilience is also viewed as a personal attribute that enables teachers to sustain their professional commitment and complete stressful tasks (Brunetti, 2006). However, it is “not primarily associated with the capacity to bounce back or recover from highly traumatic experiences and events, but rather, the capacity to maintain equilibrium and a sense of commitment and agency in the everyday worlds in which teachers teach” (Gu and Day, 2013, p. 26). Furthermore, some scholars have situated teacher resilience as a capacity or process from a social-ecological perspective (Gu, 2018; Mansfield et al., 2018; Theron, 2018) and have emphasized its function in successfully addressing daily teaching demands and maintaining their identity (Beutel et al., 2019). It can be cultivated and developed (Tait, 2008; Gu and Day, 2013; Gu, 2018) through building and activating personal and contextual resources (Day and Hong, 2016; Beutel et al., 2019). To date, research on teacher resilience has enjoyed currency, yet empirical exploration of the topic remains in its infancy (Gu and Li, 2013; Gu, 2018; Wosnitza and Peixoto, 2018), with less emphasis on language teacher resilience. From the limited research on teacher resilience conducted in the foreign language (FL) context, teacher resilience refers to teachers’ abilities to continue to function effectively in the face of teaching adversities (Kostoulas and Lämmerer, 2020). It is shaped and developed in the dynamic interaction and negotiation between teachers and the social and professional contexts in which they live (Kostoulas and Lämmerer, 2018; Fan et al., 2021). For the purpose of this study, EFL teacher resilience is conceptualized as a quality or capacity that enables teachers to recover from adversities, adapt, and thrive in their professional lives (Pretsch et al., 2012; Hiver, 2018; Kostoulas and Lämmerer, 2018, 2020; Leroux, 2018).

### Unpacking the Profiles of Teacher Resilience

Aside from the conceptualization of teacher resilience mentioned above, the empirical exploration of the topic has received some attention in educational psychology over the past two decades. Some researchers show a great willingness to examine the structure, levels, and influencing factors of teacher resilience, resilience strategies, and their relationships with other psychological variables (Howard and Johnson, 2004; Brunetti, 2006; Tait, 2008; Castro et al., 2010; Mansfield et al., 2012; Richards et al., 2016; Ellison and Woods, 2018). Connor and Davidson (2003) developed the Connor-Davidson Resilience Scale (CD-RISC), which consists of five factors: personal competence, high standards, and tenacity; trust in one’s instincts, tolerance of negative affect and strengthening effects of stress; positive acceptance of change and secure relationships; control; and spiritual influences. Yu and Zhang (2007) examined the Chinese version of the CD-RISC using a sample of 560 Chinese people (i.e., workers, teachers, and businessmen) and obtained a tri-factorial structure of resilience concerning tenacity, strength, and optimism. Later, Liu and Chu (2022) adapted the above scale and formed the CD-RISC-EFL teacher survey, which comprised three factors: tenacity, optimism, and coping style. Specifically, tenacity emphasized teachers’ perseverance and their sense of purpose and control in language teaching; optimism highlighted teachers’ confidence, positive attitudes, and acceptance in the face of pressures; and coping style focused on teachers’ dispositions toward resisting pressures and showing initiative in solving various problems. Additionally, previous empirical research examined levels of teacher resilience and its differences based on demographic variables using the resilience scale and revealed that teachers had a strong capacity for resilience to cope with challenges (Lin, 2015; Richards et al., 2016). For example, Lin (2015) identified that Chinese university teachers showed a moderately high level of resilience using the Connor-Davidson resilience scale, and male teachers were more resilient than female teachers. Bowles and Arnup (2016) found that the length of teaching experience and gender were not significantly associated with teacher resilience. Moreover, clear evidence has manifested an array of protective and risk factors affecting teacher resilience. Specifically, protective factors primarily include self-efficacy, commitment, motivation, a sense of achievement, and competence (Howard and Johnson, 2004; Beltman et al., 2011; Li et al., 2019), good student behavior, and support from leaders and families (Gu and Day, 2007; Leroux, 2018). Likewise, a large repertoire of risk factors influencing teacher resilience have also been identified, such as teacher burnout (Leroux, 2018), intention to leave (Arnup and Bowles, 2016), scarcity of resources, work–life imbalance, students’ low motivation (Gu and Day, 2013; Gu and Li, 2013), and higher requirements brought by national educational policy (Fan et al., 2021). The risk and protective factors are in a complex and dynamic relationship (Beltman et al., 2011), and multiple challenges can be perceived as opportunities for further development (Fan et al., 2021).

Resilience strategies have also been researched in the field of teacher education (Castro et al., 2010; Burns et al., 2013; Helker et al., 2018). These approaches to developing teacher resilience usually involve the use of favorable inner and external resources. For example, Castro et al. (2010) found four types of novice teacher resilience strategies: help-seeking, problem-solving, management of difficult relationships, and rejuvenation and renewal. Burns et al. (2013) identified a range of resilience strategies among university teachers with dyslexia, namely, employing task-related strategies, personalizing work contexts, utilizing social support networks, and nurturing self-esteem and self-efficacy. Overall, these studies provide feasible resilience strategies for unprivileged teachers to effectively navigate challenges in personal and professional contexts. Additionally, much attention has been given to the relationships between resilience and other variables, such as wellbeing (Pretsch et al., 2012), role stressors and burnout (Richards et al., 2016), job satisfaction and attribution (Arnup and Bowles, 2016), adaptive functioning (Bowles and Arnup, 2016), motivation (Lohbeck, 2018), and professionalism and identity (Flores, 2018). To conclude, studies on teacher resilience have gained wide currency in general education and have demonstrated diverse themes. It greatly contributes to unpacking potential factors that affect teachers’ psychological and professional growth and to discovering feasible strategies to cultivate their capacity for resilience and thrive in the school context.

Resilience, as a key factor in language teacher psychology (Mercer and Kostoulas, 2018), has drawn some attention in recent years, especially with the boom of positive psychology (Budzińska and Majchrzak, 2021; Gregersen and Mercer, 2022). In the field of foreign language teaching, to date, the emphasis has been placed on the factors affecting language teacher resilience and resilience strategies (Fan et al., 2021), resilience outcomes (i.e., adaptive or maladaptive adjustment; Kostoulas and Lämmerer, 2018, 2020), and its relationship with other variables, such as wellbeing and enjoyment (Ergün and Dewaele, 2021). For example, Kostoulas and Lämmerer (2018) created a resilience system model involving inner strengths, learned strategies, and external support, and then used semi-structured interviews to elucidate the development of resilience of a language teacher during a period of career transition. Ergün and Dewaele (2021) found that Italian as a Foreign Language teachers exhibited high levels of resilience independent of gender, age, and length of experience of resilience. The authors also highlighted that resilience and wellbeing served as significant predictors of teaching enjoyment. In summary, empirical research on language teacher resilience is still lacking and can be expanded further in different educational settings. Moreover, little has been done in China in this regard (Fan et al., 2021; Liu and Chu, 2022; Wang et al., 2022), especially using quantitative or mixed research methods. However, Chinese EFL teachers are also confronted with a series of risks, such as a dearth of confidence in language proficiency, large-class teaching, inadequate teaching resources, and educational reform (Liu and Li, 2020; Wen and Zhang, 2020). This is accentuated in the case of EFL teachers working in Chinese senior high schools, as they experience intense teaching pressure driven by the national college entrance examination, which is regarded as a turning point in student academic development (Chu et al., 2021). Additionally, there are different voices about the links between demographic variables (i.e., gender, educational background, and length of experience) and teacher resilience (Lin, 2015; Kostoulas and Lämmerer, 2020; Ergün and Dewaele, 2021), which have not been thoroughly examined in the FL-specific domain. The exploration of EFL teacher resilience helps us understand how they overcome multiple challenges and further offers targeted strategies to sustain their psychological and professional development in stressful contexts (Fan et al., 2021; Zhang, 2021). In this context, the current study, as part of the pilot study^1^ of a larger project (see the acknowledgements), aims to adopt a mixed-methods design to examine EFL teacher resilience in China by addressing the following research questions:

1. What are the levels of Chinese EFL teacher resilience?
2. What are the differences in Chinese EFL teacher resilience with regard to demographic variables (i.e., gender, years of teaching experience, and educational background)?

## Methodology

An explanatory sequential mixed-method design was utilized in this study, which involved collecting quantitative data first and then explaining the quantitative results with in-depth qualitative data (Creswell and Clark, 2017). Specifically, we employed quantitative data to examine the levels of EFL teacher resilience in the Chinese context and its differences in light of demographic factors. We subsequently used qualitative data to unravel the quantitative results.

### Research Participants

Liu and Chu (2022) confirmed a tri-factorial structure of EFL teacher resilience based on a sample of 330 teachers. The current study was conducted among these 330 teachers (300 were female and 30 were male). These participants showed different years of teaching experience from no more than 5 years (*n* = 86), 5–10 years (*n* = 62), 10–20 years (*n* = 106), and more than 20 years (*n* = 76). Among these participated teachers, 258 of them had bachelor’s degrees, and 72 had master’s degrees.

We also invited five participants to join the interview part of the current study from 34 respondents who filled in their positive answers to the last item (Whether you voluntarily participate in the follow-up interview) of the questionnaire. In selecting the interviewees, we set up the following criteria: both male and female EFL teachers were involved; teachers possessed bachelor’s or master’s degrees; they were situated in different career stages; they showed great willingness to share their learning and teaching experience (see Table 1).

**Table 1:** Basic information of the five interviewes.

Basic Information	Teacher A	Teacher B	Teacher C	Teacher D	Teacher E
Gender	Female	Female	Female	Male	Male
Years of teaching experience	3	8	8	13	26
Educational degree	Master	Bachelor	Master	Bachelor	Bachelor
Length of interview	40 min	53 min	42 min	49 min	45 min

### Research Instruments

Two instruments were used in this study: the Connor-Davidson Resilience Scale (CD-RISC)-EFL Teacher Survey and semi-structured interviews. The CD-RICS-EFL Teacher Survey (Liu and Chu, 2022) was adapted from the original version of the CD-RISC (Connor and Davidson, 2003) and the Chinese version of the CD-RISC (Yu and Zhang, 2007). The validated version by Liu and Chu (2022) included three dimensions: tenacity, optimism, and coping style. Tenacity refers to teachers’ perseverance and their sense of purpose and control in language teaching. Optimism refers to teachers’ confidence, positive attitudes, and acceptance in the face of pressures and challenges. Coping style, as a newly emerged dimension, refers to EFL teachers’ dispositions toward resisting pressures and showing initiative in navigating multiple risks. Responses were scored on a five-point Likert scale ranging from 1 (not true at all) to 5 (true all the time). This validated 10-item scale showed acceptable reliability (Cronbach’s alpha coefficients of global resilience to sub-dimensions ranged from 0.783 to 0.662, see Table 2) and good structural validity (see Liu and Chu, 2022).

**Table 2:** Basic information from the CD-RISC-EFL Teacher Survey.

Dimension	Item no.	*ɑ*	Sample items
Tenacity	11, 21, 22	0.662	21: Strong sense of purpose in English teaching
Optimism	01, 02, 04, 06	0.706	06: See the humorous side of things
Coping Style	15, 18, 20	0.765	15: Prefer to take the lead in English teaching problem solving
Global Resilience	—	0.783	—

An interview protocol was employed to conduct the semi-structured interviews, which was adopted to gather more information about the challenges that EFL teachers have encountered and their attitudes and strategies in the face of those adversities. The following questions were focused on in the interviews:

1. What do you think of the English language teaching profession?
2. Are there any stressful events in your teaching or personal life?
3. How do you feel about and deal with those challenges?
4. Are there any resources available that help you address these challenges?

### Data Collection

Electronic questionnaires were given to participants from September to October 2019 through an online questionnaire system. Before completing the survey, the participants were informed about the research purposes and assured that their personal information would be kept confidential. Semi-structured interviews were conducted with those voluntary EFL teachers for further analysis. The interviews were conducted in Putonghua for 45 min on average and audio-recorded with the participants’ permission.

### Data Analysis

All quantitative data were recorded and analyzed using SPSS 26.0. We adopted descriptive statistics, independent-samples *t*-tests, and one-way ANOVA to explore the levels and demographic variation of EFL teacher resilience. In addition, qualitative content analysis was employed to analyze the interview data (Dörnyei, 2007; Miles et al., 2014). The qualitative interview data were first transcribed in textual form by the researchers and checked by the interviewees, and then it was coded after iterative reading and reflection. It allowed researchers to develop the ideas, interpret data, and draw conclusions, and thus triangulate and enrich the quantitative results in this study.

## Results

### General Description of EFL Teacher Resilience

Table 3 presents the details of the levels of Chinese EFL teacher resilience at both the global and dimensional levels. Using the five-point Likert scale employed in the current study, it was found that EFL teachers displayed a moderate to high level of resilience (*M* = 3.80, *SD* = 0.44) in general. As for the three factors of resilience, EFL teachers had a higher level of tenacity (*M* = 4.01, *SD* = 0.47), followed by optimism (*M* = 3.84, *SD* = 0.46) and coping style (*M* = 3.57, *SD* = 0.62).^2^

**Table 3:** The results of the descriptive analysis of English as a foreign language (EFL) teacher resilience.

Dimension	Min	Max	*M*	SD
Tenacity	2.67	5.00	4.01	0.47
Optimism	2.25	5.00	3.84	0.46
Coping style	2.00	5.00	3.57	0.62
Global resilience	2.58	5.00	3.80	0.44

As revealed in Table 3, senior high school English teacher resilience was at a moderate to high level (*M* = 3.80, *SD* = 0.44), which indicated that teachers had the capacity to cope with teaching challenges and were adaptable in their personal and professional lives. Qualitative interview data were also obtained to explicate this quantitative result, which are shown in the following extracts:

Extract 1

*To date, there has been no very serious challenge for me. The only difficult thing was poor classroom discipline, but I could try to maintain a harmonious class atmosphere. Overall, students usually performed well under my supervision. (Teacher A, 2019/09/30)*

Extract 2

*I once encountered a period of professional burnout and felt confused, so I talked with the experienced teachers and sought suggestions. They shared their learning and teaching experiences and told me how to get along with students and improve teaching skills. Later, I positively engaged in some teaching competitions and went out for further studies. As a teacher, I could not stamp on the same ground all the time and needed continuous learning. (Teacher B, 2019/09/28)*

Extract 3

*What most troubled me was that students were not interested in English, especially in the low-level class, but I tried to find topics close to their daily life, and asked experienced teachers for help.… I got a sense of accomplishment and happiness, as my students started to listen to class carefully. (Teacher C, 2019/09/27)*

As these excerpts revealed, an increasing number of teaching challenges appeared in the professional world of these EFL teachers, as Teachers A and C mentioned students’ low learning motivation, misbehavior, and poor classroom discipline, and Teacher B highlighted the risk of professional burnout in the school context. Some teachers felt depressed and confused in the presence of teaching risks, but were not easily demoralized, and still attempted to engage students in English language classrooms or seek their professional development. For example, Teacher B positively sought support and advice from veteran teachers and showed a great willingness to develop her teaching skills through participating in teaching competitions and further studies. These findings were also reflected in Liu and Chu’s (2022) study, which highlighted that teachers, as active agents situated in the educational context, could positively negotiate with their surroundings and exert their agency (Huang, 2021; Liu et al., 2022) to cope with professional and personal pressures, thus progressively shaping and developing their capacity for resilience. Additionally, the data analysis also implied that teachers gained a sense of wellbeing and strengthened their professional identity while responding to adversities.

### Gender Differences in EFL Teacher Resilience

In order to mitigate the effects caused by gender imbalance in the sample (Male = 30, Female = 300), the authors randomly selected about 10% of female teachers using SPSS software and constructed a new sample (Male = 30, Female = 37). According to the data in Table 4, there was no significant difference in resilience between male and female EFL teachers (*t* = 0.550; *df* = 65; *p* = 0.584). On the whole, both male and female EFL teacher resilience were at a moderate to high level.

**Table 4:** The independent-samples *t*-test of EFL teacher resilience on gender.

Dimension	Male (*n* = 30)		Female (*n* = 37)		*MD*	*t*	*Sig.*
	*M*	*SD*	*M*	*SD*			
Tenacity	3.93	0.49	3.88	0.54	0.05	0.396	0.693
Optimism	3.79	0.58	3.76	0.54	0.03	0.254	0.800
Coping style	3.51	0.55	3.40	0.67	0.11	0.756	0.452
Global resilience	3.75	0.45	3.69	0.49	0.06	0.550	0.584

The results in Table 4 imply that there was no significant difference in EFL teacher resilience attributable to gender. In other words, both male and female teachers were able to face challenges and endeavor to tackle stressful events. However, some differences between female and male teachers in navigating risks also exist. As illustrated in the following interview extracts:

Extract 4

*I was busy with my work, but my children were young. I needed to spend much time taking care of them and balancing my work and family life…. Actually, female teachers experienced more pressure in the daily life and male teachers were more relaxed. (Teacher B, Female, 2019/09/28)*

Extract 5

*I once had a conflict with students’ parents due to students’ misbehavior, and the school criticized me first…. I talked about it with my family. They told me that I should communicate with them sincerely. Teaching was a matter of dedication, and I needed to solve practical problems for them. Later, I started to reflect on myself and tackled those challenges smoothly. (Teacher C, Female, 2019/09/27)*

Extract 6

*My daughter went to school at 6:00 AM and arrived home at 6:00 PM. I had no time to give her any guidance in her studies. Recently, my little son was sick, and my wife took him to the hospital. I felt sad and guilty because I could not take time off from work to look after them…. A man experienced some challenges and then grew up. Despite various setbacks, I could not always complain to my family and students. (Teacher D, Male, 2019/10/17)*

From the above interviews, it was found that male and female teachers both faced pressures arising from the school and family, such as work–life imbalance, teacher–school conflicts, and misunderstandings from students’ parents. However, they were never overwhelmed by the array of teaching and personal challenges; instead, they were resilient and displayed an earnest attitude toward teaching tasks. It is worth noting that male and female teachers utilized different approaches while dealing with those challenges. For example, Teacher D (Male) was inclined to bear hardships without complaining and to control his negative emotions when getting along with students and families. In contrast, female teachers were more willing to communicate with and seek support from important people in their lives (i.e., family members, colleagues, and school leaders).

### Differences in Years of Teaching Experience in EFL Teacher Resilience

The data analysis in this section attempts to answer the research question, “What are the differences in EFL teacher resilience with regard to years of teaching experience?” YTE represents years of teaching experience, which is divided into four categories: *Y*_1_ ≤ 5, 5 < *Y*_2_ ≤ 10, 10 < *Y*_3_ ≤ 20, 20 < *Y*_4_. As indicated in Table 5, there was no significant difference in resilience of EFL teachers attributable to years of teaching experience [*F*(3,326) = 0.437, *p* > 0.05]. Additionally, no significant difference in three factors (tenacity, optimism, and coping style) was found among those teachers with different teaching years (*p* > 0.05).

**Table 5:** One-way ANOVA of EFL teacher resilience on years of teaching experience.

Dimension	*Y*_1_ (*n* = 86)		*Y*_2_ (*n* = 62)		*Y*_3_ (*n* = 106)		*Y*_4_ (*n* = 76)		*F*	Sig.
	*M*	*SD*	*M*	*SD*	*M*	*SD*	*M*	*SD*		
Tenacity	4.06	0.45	3.96	0.36	4.00	0.40	3.98	0.68	0.376	0.770
Optimism	3.94	0.43	3.76	0.41	3.85	0.46	3.76	0.55	1.677	0.173
Coping style	3.57	0.59	3.66	0.55	3.46	0.65	3.68	0.66	1.413	0.240
Global resilience	3.86	0.39	3.79	0.35	3.77	0.42	3.80	0.58	0.437	0.727

Overall, senior high school EFL teachers with different teaching years believed that they had the capacity to handle challenges. In particular, they displayed a relatively high level of tenacity and emphasized their sense of purpose and control in language teaching. Furthermore, early-career EFL teachers seemed to be more optimistic than experienced teachers, but the latter performed better in coping style. The interview data, to a certain degree, resonated with this quantitative result:

Extract 7

*I thought something like lesson preparation and school meetings might decrease my sense of well-being, but it was really enjoyable to teach students and get along with them. (Teacher A, 3 YTE, 2019/09/30)*

Extract 8

*Some students might have diverged thinking abilities or feel close to me, and they liked to ask me some funny questions and attract my attention. Under these circumstances, I tended to guide them to perform well in class rather than damage their confidence, but sometimes I also got emotional or angry due to their misbehavior. (Teacher C, 8 YTE, 2019/09/27)*

Extract 9

*The competitions between teachers were also very stressful, and students were getting smarter…. I thought I should perform like a hardworking student, try to learn more new things and update my knowledge and skills. (Teacher D, 13 YTE, 2019/10/17)*

Extract 10

*While suffering from many challenges caused by students, I learned to control my negative emotions, and I told myself that I was a teacher and should become more sensible and calmer. (Teacher E, 26 YTE, 2019/10/21)*

English as a foreign language teachers were situated in school environments and encountered similar professional pressures in spite of the difference in years of teaching experience. As stated earlier, challenges from competition among teachers, students’ misbehavior, time-consuming lesson preparation, and school meetings seemed to propose higher demands for teaching staff, thus pushing them to respond to these adversities. Specifically, younger teachers (i.e., Teacher A) showed a more optimistic attitude toward EFL teaching and students and emphasized that stressful events that could be tackled were not viewed as setbacks. However, sometimes they might get emotional in the presence of students’ poor performance. Unlike young teachers, teachers with more teaching experience (i.e., Teachers D and E) were inclined to regulate their own emotions and reflect on their teaching performance. Moreover, it might become an important agenda for veteran teachers to update their teaching knowledge and skills in a timely manner. Overall, EFL teachers in different career stages showed their distinctive abilities (i.e., active reflection, emotion regulation, a sense of competence, and wellbeing), endeavored to solve teaching problems, and served students better in the school context.

### Differences in Educational Background in EFL Teacher Resilience

The independent samples *t*-test was used to investigate whether there was a significant difference in resilience between teachers with different educational backgrounds. According to the data available, the participants’ educational backgrounds can be divided into two degrees: bachelor and master. To reduce the effects caused by the imbalance of educational background in the sample (Bachelor = 258, Master = 72), the authors randomly selected about 30% of teachers with only bachelor’s degrees using SPSS software and created a new sample (Bachelor = 71, Master = 72). As shown in Table 6, the results revealed that there was a slight difference in resilience among EFL teachers with different educational backgrounds (*t* = −1.993; *df* = 141; *p* < 0.05). More precisely, EFL teachers with master’s degrees (*M* = 3.98, *SD* = 0.42) possessed a higher level of resilience than those with only bachelor’s degrees (*M* = 3.78, *SD* = 0.44). Additionally, among the three dimensions of resilience, a significant difference in tenacity and optimism was found between teachers with different degrees (*p* < 0.05), but there was no significant difference with regard to coping style (*p* > 0.05).

**Table 6:** The independent-samples *t*-test of EFL teacher resilience on educational background.

Dimension	Bachelor (*n* = 71)		Master (*n* = 72)		*MD*	*t*	Sig.
	*M*	*SD*	*M*	*SD*			
Tenacity	3.98	0.46	4.25	0.54	−0.27	−2.248	0.011
Optimism	3.81	0.46	4.06	0.43	−0.25	−2.346	0.020
Coping style	3.56	0.62	3.63	0.66	−0.07	−0.533	0.595
Global resilience	3.78	0.44	3.98	0.42	−0.20	−1.993	0.048

Although teachers with different educational degrees displayed a moderately high level of resilience in general, teachers with master’s degrees were more resilient. They showed more positive attitudes toward teaching challenges and had a stronger sense of control and competence in English language teaching. This might be observed in the following extracts:

Extract 11

*When school leaders listened to my class, I felt stressed and solemn. There was no big change in the whole teaching process, but perhaps my students might perform better than before…. I also thought about how to make my lectures more interesting and effective. (Teacher B, Bachelor, 2019/09/28)*

Extract 12

*School leaders sometimes checked teachers’ lesson plan*s *or listened to your classes suddenly, which might have been an inspection of our working attitudes… I was stressed, especially when I was not prepared well. However, I would adjust my mood and try to design more attractive teaching activities. (Teacher D, Bachelor, 2019/10/17)*

Extract 13

*I really liked attracting people’s attention in my class. It pushed me to continuously improve myself and motivated my students to engage positively in teaching activities, especially when leaders required me to do some demonstration classes. I obtained some useful feedback from experts. (Teacher A, Master, 2019/09/30)*

Extract 14

*I usually communicated with my colleagues after class and sought their feedback on my teaching performance. Then, I might modify my teaching contents or methods for effectively teaching English. I also liked to listen to the lessons of my colleagues, especially those experienced teachers. (Teacher C, Master, 2019/09/27)*

The above interviews suggested that EFL teachers with master’s degrees seemed to show more positive acceptance of changes or pressures and attempted to seek external resources (e.g., open class, feedback from experts or leaders, and class observation) to promote their professional development. However, EFL teachers with bachelor’s degrees aimed to complete the teaching tasks smoothly and perform well in front of school leaders. Simultaneously, they (i.e., Teachers B and D) also engaged in continuous reflection and exploration and hoped to make teaching more effective and energetic. Taken together, they were able to adapt to a challenging teaching environment and develop themselves well.

## Discussion

In general, EFL teachers showed moderate to high levels of resilience, indicating that teachers had some capacities that enabled them to recover from adversities, adapt, and thrive in their personal and professional lives. This result was partly supported by the findings of Richards et al. (2016), highlighting teachers’ moderately high levels of resilience and the positive role of resilience in avoiding negative consequences and reducing teachers’ perceived stress and burnout. There are several possible explanations for the moderate to high levels of resilience exhibited by EFL teachers. First, priority should be given to teachers’ personal strengths that contribute to developing teacher resilience, such as self-efficacy, professional identity, commitment to, and love for the teaching profession (Gu and Day, 2007; Gu and Li, 2013; Kostoulas and Lämmerer, 2018, 2020; Li et al., 2019; Fan et al., 2021; Gong et al., 2021). EFL teachers with stronger self-efficacy showed more positive perceptions of teachers’ capacities to complete English teaching tasks, successfully impart knowledge, and strategically engage students in classroom activities (Liu et al., 2021) and were willing to exert their agency to manage various teaching adversities (Huang, 2021; Huang and Yip, 2021). Interview data also revealed that some EFL teachers (i.e., Teachers A and C) showed love for their job and higher professional identity, and they viewed teaching as a challenging but happy profession. They were driven by their teaching beliefs, showed a great willingness to invest substantial energy and time in promoting students’ academic achievement, and simultaneously gained emotional rewards from teaching practice (Chu et al., 2021). Unsurprisingly, EFL teachers became more resilient and taught students to the best of their abilities in complicated school settings. Second, support from important figures has also contributed to building and sustaining teacher resilience (Day and Hong, 2016; Li et al., 2019; Liu and Chu, 2022). Interview extracts (i.e., Teachers A, B, and C) also revealed that harmonious interpersonal relationships with colleagues and students gave teachers a sense of wellbeing and motivated them to smoothly cope with teaching activities. Third, the English language was an essential subject in the Chinese educational system, and students’ academic performance in senior high schools manifested through the national college entrance examination (Wen and Zhang, 2020; Chu et al., 2021; Liu and Chu, 2022). School administrators paid adequate attention to EFL teachers in Chinese senior high schools and encouraged them to teach students to the best of their abilities. These favorable resources provided by the educational institution could foster teachers to enact their agency in navigating a variety of teaching challenges (Huang, 2021), thus strengthening their capacity for resilience.

Three salient and oft-discussed demographic variables (gender, years of teaching experience, and educational background) were selected to examine their relationships with resilience. According to the data analysis, there was no significant difference in resilience between male and female EFL teachers. This result was similar to previous findings (Lin, 2015; Kostoulas and Lämmerer, 2020) that indicated no statistically significant links related resilience to variables concerning age and gender. The study also suggested that some differences between female and male teachers in navigating risks also exist, while both male and female EFL teachers displayed their capacity for resilience. The interview excerpts revealed that male teachers usually bore responsibility and navigated challenges by themselves, and they seemed to be more perseverant in the presence of pressure. It might be related to the fact that men were endowed with more responsibility, tenacity, and strength in the Chinese cultural context (Lin, 2015). Furthermore, EFL teachers with different teaching years yielded a similar level of resilience, and years of teaching experience had no significant effect on resilience (Ergün and Dewaele, 2021). Indeed, regardless of the duration of their experience, teachers might be more affected by their other distinctive qualities (i.e., professional identity, commitment, and self-efficacy) and sociocultural factors (Gu, 2018; Chu et al., 2021; Liu and Chu, 2022), especially when they shared common teaching goals (i.e., educating students and good academic performance in the national college entrance examination). In addition, the data analysis indicated a significant difference in resilience between EFL teachers with bachelor’s and master’s degrees. EFL teachers with master’s degrees presented a slightly higher level of resilience. This might be closely pertinent to their EFL learning and teaching experiences. The interviews with Teachers A and C highlighted that they were trained in how to teach and manage students effectively and received much feedback from teachers in the demonstration classes when they pursued their master’s degrees. Previous research showed that continuous reform for language education in China was implemented and called for the cultivation of more qualified teachers (Gao and Xu, 2014; Wen and Zhang, 2020; Chu et al., 2021). For example, Liu and Li (2020) pointed out that the Foreign Teacher Education-Master’s Degree of Education (FTE-MAEd) program launched by the Ministry of Education of China became a feasible approach to retain teachers and motivate novice teachers to engage in continuing professional development as part-time MAEd students. Therefore, to a certain extent, EFL teachers with master’s degrees had more opportunities afforded by a supportive school context to learn from experts and gain advanced teaching ideas and methods, thus building their stronger capacity for resilience.

There were cohorts of risk factors, such as bad classroom discipline, students’ misbehavior, heavy workloads, long working hours, and competition among teachers, which affected the psychological and physical development of EFL teachers. Nevertheless, teachers with unique histories, personalities, identities, and motives (Ushioda, 2020), could exercise their agency, take potential approaches to avoid or respond to these challenges (Chu et al., 2021; Huang, 2021; Liu et al., 2021), and grow into more resilient persons. Furthermore, teaching was a relatively stable profession in Chinese educational contexts (Gu and Li, 2013; Li et al., 2019), and some national policies related to EFL teachers’ professional development were implemented and attempted to nurture qualified teachers and improve their working conditions (Gao and Xu, 2014; Liu and Li, 2020). Multiple internal and external protective resources have greatly contributed to the promotion of EFL teacher resilience and quality retention.

## Conclusion

The current study investigated EFL teacher resilience in the Chinese context using a mixed-methods design. It was found that Chinese EFL teachers in senior high schools showed moderately high levels of resilience. Meanwhile, no significant difference was found between EFL teachers and the demographic factors of gender and years of teaching experience, but there was a slight statistically significant difference between the resilience of teachers with different educational backgrounds. Compared with senior high school teachers with only bachelor’s degrees, teachers with master’s degrees exhibited a higher level of resilience. Notably, these EFL teachers, as developing persons situated in ecological environments, were able to respond actively to multiple challenges and promote their capacity for resilience.

The findings of this study have several pedagogical implications for developing EFL teacher resilience. First, EFL teachers should have clear self-awareness and fully employ their personal resources in the face of adversities. There are many distinctive qualities exhibited by EFL teachers, such as a spirit of dedication, love for teaching, stronger self-efficacy, and identity (Chu et al., 2021; Fan et al., 2021; Liu et al., 2021). These teacher-related protective factors are conducive to avoiding or addressing some risks and thus developing teacher resilience (Mansfield et al., 2016; Richards et al., 2016). Second, teachers shape and sustain their resilience through a web of trusting interpersonal relationships (Gu, 2018; Li et al., 2019). EFL teachers of different genders, teaching experiences, and educational backgrounds can strengthen cooperation with each other, simultaneously encourage more communication with leaders and experts and seek support from their social networks when faced with complex teaching challenges (Fan et al., 2021). This study also has some limitations that suggest directions for future research. Specifically, the interview and questionnaire sample sizes were slightly small, and there were imbalances in the sample in terms of gender and educational background. It is recommended that future studies enlarge the scope and sample, and fully consider the balance of samples in terms of demographic information. Hopefully, the adapted 10-item CD-RISC-EFL Teacher Survey can serve as a useful instrument to examine EFL teacher resilience in other contexts and its relationships with other variables, such as teacher engagement, motivation, professional identity, and commitment, through a large-scale sample.

## Data Availability Statement

The raw data supporting the conclusions of this article will be made available by the authors, without undue reservation.

## Ethics Statement

The studies involving human participants were reviewed and approved by School of Foreign Languages, Northeast Normal University, China. Written informed consent to participate in this study was provided by the participants’ legal guardian/next of kin. Written informed consent was obtained from the individual(s) for the publication of any potentially identifiable images or data included in this article.

## Author Contributions

WC conceptualization, data analysis, writing, and revision. HL conceptualization, data analysis, revision, supervision, and funding. All authors contributed to the article and approved the submitted version.

## Funding

The research was supported by the National Social Science Fund of China (Grant number 21BYY120) as part of the project entitled *Research on the Foreign Language Teacher Resilience: The Ecology of Human Development Perspective.*

## Conflict of Interest

The authors declare that the research was conducted in the absence of any commercial or financial relationships that could be construed as a potential conflict of interest.

## Publisher’s Note

All claims expressed in this article are solely those of the authors and do not necessarily represent those of their affiliated organizations, or those of the publisher, the editors and the reviewers. Any product that may be evaluated in this article, or claim that may be made by its manufacturer, is not guaranteed or endorsed by the publisher.

## Footnotes

^1^This pilot study entitled *An Empirical Study of Senior High School English Teacher Resilience* was conducted by the first author and supervised by the second author of the paper. Please contact the first author for details of the pilot study.

^2^Findings reported in Table 3 were discussed in Liu and Chu (2022), but the interview data only appeared in the current paper.

## References

[ref1] Acheson K. Nelson R. (2020). “Utilising the emotional labour scale to analyse the form and extent of emotional labour among foreign language teachers in the US public school system,” in The Emotional Rollercoaster of Language Teaching. eds. Gkonou C. Dewaele J.-M. King J. (Bristol: Multilingual Matters), 31–52.

[ref2] Arnup J. Bowles T. (2016). Should I stay or should I go? Resilience as a protective factor for teachers’ intention to leave the teaching profession. Aust. J. Educ. 60, 229–244. doi: 10.1177/0004944116667620

[ref3] Beltman S. Mansfield C. Price A. (2011). Thriving not just surviving: a review of research on teacher resilience. Educ. Res. Rev. 6, 185–207. doi: 10.1016/j.edurev.2011.09.001

[ref4] Beutel D. Crosswell L. Broadley T. (2019). Teaching as a ‘take-home’ job: understanding resilience strategies and resources for career change preservice teachers. Aust. Educ. Res. 46, 607–620. doi: 10.1007/s13384-019-00327-1

[ref5] Bowles T. Arnup J. L. (2016). Early career teachers’ resilience and positive adaptive change capabilities. Aust. Educ. Res. 43, 147–164. doi: 10.1007/s13384-015-0192-1

[ref6] Brunetti G. J. (2006). Resilience under fire: perspectives on work of experienced, inner city high school teachers in the United States. Teach. Teach. Educ. 22, 812–825. doi: 10.1016/j.tate.2006.04.027

[ref7] Budzińska K. Majchrzak O. (2021). Positive Psychology in Second and Foreign Language Education. Cham: Springer.

[ref8] Burns E. Poikkeus A. M. Aro M. (2013). Resilience strategies employed by teachers with dyslexia working at tertiary education. Teach. Teach. Educ. 34, 77–85. doi: 10.1016/j.tate.2013.04.007

[ref9] Castro A. J. Kelly J. Shih M. (2010). Resilience strategies for new teachers in high-needs areas. Teach. Teach. Educ. 26, 622–629. doi: 10.1016/j.tate.2009.09.010

[ref10] Chu W. Liu H. Fang F. (2021). A tale of three excellent Chinese EFL teachers: unpacking teacher professional qualities for their sustainable career trajectories from an ecological perspective. Sustain. For. 13:6721. doi: 10.3390/su13126721

[ref11] Connor K. M. Davidson J. R. T. (2003). Development of a new resilience scale: the Connor-Davidson resilience scale (CD-RISC). Depress. Anxiety 18, 76–82. doi: 10.1002/da.10113, PMID: 12964174

[ref12] Creswell J. W. Clark V. L. P. (2017). Designing and Conducting Mixed Methods Research (3rd Edn.). California: Sage Publications.

[ref13] Day C. Hong J. (2016). Influences on the capacities for emotional resilience of teachers in schools serving disadvantaged urban communities: challenges of living on the edge. Teach. Teach. Educ. 59, 115–125. doi: 10.1016/j.tate.2016.05.015

[ref14] Dewaele J.-M. (2020). “What psychological, linguistic and sociobiographical variables power EFL/ESL teachers’ motivation,” in The Emotional Rollercoaster of Language Teaching. eds. Gkonou C. Dewaele J.-M. King J. (Bristol: Multilingual Matters), 269–287.

[ref15] Dörnyei Z. (2007). Research Methods in Applied Linguistics: Quantitative, Qualitative and Mixed Methodologies. New York: Oxford University Press.

[ref16] Ellison D. W. Woods A. M. (2018). Physical education teacher resilience in high-poverty school environments. Eur. Phys. Educ. Rev. 25, 1110–1127. doi: 10.1177/1356336X18800091

[ref17] Ergün A. L. P. Dewaele J.-M. (2021). Do well-being and resilience predict the foreign language teaching enjoyment of teachers of Italian? System 99:102506. doi: 10.1016/j.system.2021.102506

[ref18] Fan L. Ma F. Liu Y. Liu T. Guo L. Wang L. (2021). Risk factors and resilience strategies: voices from Chinese novice foreign language teachers. Front. Educ. 5:565722. doi: 10.3389/feduc.2020.565722

[ref19] Fenton-O’Creevy M. Dimitriadis Y. Scobie G. (2015). “Failure and resilience at boundaries: the emotional process of identity work,” in Learning in Landscapes of Practice. eds. Wenger-Trayner E. Fenton-O’Creevy M. Hutchinson S. Kubiak C. Wenger-Trayner B. (London: Routledge), 33–42.

[ref20] Flores M. A. (2018). “Teacher resilience in adverse contexts: Issues of professionalism and professional identity,” in Resilience in Education: Concepts, Contexts and Connections. eds. Wosnitza M. Peixoto F. Beltman S. Mansfield C. F. (Cham: Springer), 167–184.

[ref21] Gao X. Xu H. (2014). The dilemma of being English language teachers: interpreting teachers’ motivation to teach, and professional commitment in China's hinterland regions. Lang. Teach. Res. 18, 152–168. doi: 10.1177/1362168813505938

[ref22] Gkonou C. Dewaele J.-M. King J. (2020). The Emotional Rollercoaster of Language Teaching. Bristol: Multilingual Matters.

[ref23] Gong Y. F. Lai C. Gao X. (2021). Language teachers’ identity in teaching intercultural communicative competence. Lang. Cult. Curric. 1–17. doi: 10.1080/07908318.2021.1954938 PMC9366292 35967630

[ref24] Gregersen T. Mercer S. (2022). The Routledge Handbooks of the Psychology of Language Learning and Teaching. New York and London: Routledge.

[ref25] Gu Q. (2018). “(Re)conceptualising teacher resilience: a social-ecological approach to understanding teachers’ professional worlds,” in Resilience in Education: Concepts, Contexts and Connections. eds. Wosnitza M. Peixoto F. Beltman S. Mansfield C. F. (Cham: Springer), 13–33.

[ref26] Gu Q. Day C. (2007). Teachers’ resilience: a necessary condition for effectiveness. Teach. Teach. Educ. 23, 1302–1316. doi: 10.1016/j.tate.2006.06.006

[ref27] Gu Q. Day C. (2013). Challenges to teacher resilience: conditions count. Br. Educ. Res. J. 39, 1–23. doi: 10.1080/01411926.2011.623152

[ref28] Gu Q. Li Q. (2013). Sustaining resilience in times of change: stories from Chinese teachers. Asia Pac. J. Teach. Educ. 41, 288–303. doi: 10.1080/1359866X.2013.809056

[ref29] Helker K. Mansfield C. F. Wosnitza M. Stiller H. (2018). “An exploratory interview study of university teacher resilience,” in Resilience in Education: Concepts, Contexts and Connections. eds. Wosnitza M. Peixoto F. Beltman S. Mansfield C. F. (Cham: Springer), 185–201.

[ref30] Hiver P. (2018). “Teachstrong: The power of teacher resilience for second language practitioners,” in Language Teacher Psychology. eds. Mercer S. Kostoulas A. (Bristol: Multilingual Matters), 231–246.

[ref31] Howard S. Johnson B. (2004). Resilient teachers: resisting stress and burnout. Soc. Psychol. Educ. 7, 399–420. doi: 10.1007/S11218-004-0975-0

[ref32] Huang J. (2021). Sustainability of professional development: a longitudinal case study of an early career ESL teacher’s agency and identity. Sustain. For. 13:9025. doi: 10.3390/su13169025

[ref33] Huang J. Yip J. W. C. (2021). Understanding ESL teachers’ agency in their early years of professional development: a three-layered triadic reciprocity framework. Front. Psychol. 12:739271. doi: 10.3389/fpsyg.2021.739271, PMID: 34566821 PMC8456001

[ref34] Kidger J. Brockman R. Tilling K. Campbell R. Ford T. Araya R.. (2016). Teachers’ wellbeing and depressive symptoms, and associated risk factors: a large cross sectional study in English secondary schools. J. Affect. Disord. 192, 76–82. doi: 10.1016/j.jad.2015.11.054, PMID: 26707351

[ref35] Kostoulas A. Lämmerer A. (2018). “Making the transition into teacher education: resilience as a process of growth,” in Language Teacher Psychology. eds. Mercer S. Kostoulas A. (Bristol: Multilingual Matters), 247–263.

[ref36] Kostoulas A. Lämmerer A. (2020). “Resilience in language teaching: adaptive and maladaptive outcomes in pre-service teachers,” in The Emotional Rollercoaster of Language Teaching. eds. Gkonou C. Dewaele J.-M. King J. (Bristol: Multilingual Matters), 89–110.

[ref37] Leroux M. (2018). “Exploring Canadian early career teachers’ resilience from an evolutionary perspective,” in Resilience in Education: Concepts, Contexts and Connections. eds. Wosnitza M. Peixoto F. Beltman S. Mansfield C. F. (Cham: Springer), 107–129.

[ref38] Li Q. Gu Q. He W. (2019). Resilience of Chinese teachers: why perceived work conditions and relational trust matter. Meas. Interdiscip. Res. Perspect. 17, 143–159. doi: 10.1080/15366367.2019.1588593

[ref39] Lin X. (2015). Relationship of university teachers’ mental resilience and work stress. Univ. Educ. Sci. 4, 74–79.

[ref40] Liu H. Chu W. (2022). Exploring EFL teacher resilience in the Chinese context. System 105:102752. doi: 10.1016/j.system.2022.102752

[ref41] Liu H. Chu W. Wang Y. (2021). Unpacking EFL teacher self-efficacy in livestream teaching in the Chinese context. Front. Psychol. 12:717129. doi: 10.3389/fpsyg.2021.717129, PMID: 34421768 PMC8374736

[ref42] Liu H. Li Z. (2020). “High school teacher retention” in Exploring Teacher Recruitment and Retention. eds. Ovenden-Hope T. Passy R. (London: Routledge), 176–184.

[ref100] Liu H. Yan C. Fu J. (2022). Exploring livestream English teaching anxiety in the Chinese context: An ecological perspective. Teach. Teach. Educ. 111, 1–10. doi: 10.1016/j.tate.2021.103620

[ref200] Lohbeck L. (2018). “The interplay between the motivation to teach and resilience of student teachers and trainee teachers,” in Resilience in Education: Concepts, Contexts and Connections. eds. Wosnitza M. Peixoto F. Beltman S. Mansfield C. F. (Cham: Springer), 93–106.

[ref43] Luthar S. S. Cicchetti D. (2000). The construct of resilience: implications for interventions and social policies. Dev. Psychopathol. 12, 857–885. doi: 10.1017/S0954579400004156, PMID: 11202047 PMC1903337

[ref44] MacIntyre P. D. Ross J. Talbot K. Mercer S. Gregersen T. Banga C. A. (2019). Stressors, personality and wellbeing among language teachers. System 82, 26–38. doi: 10.1016/j.system.2019.02.013

[ref300] Mansfield C. F. Beltman S. Broadley T. Weatherby-Fell N. (2016). Building resilience in teacher education: An evidenced informed framework. Teach. Teach. Educ. 54, 77–87. doi: 10.1016/j.tate.2015.11.016

[ref45] Mansfield C. F. Beltman S. Price A. McConney A. (2012). Don’t sweat the small stuff: understanding teacher resilience at the chalkface. Teach. Teach. Educ. 28, 357–367. doi: 10.1016/j.tate.2011.11.001

[ref46] Mansfield C. F. Ebersöhn L. Beltman S. Loots T. (2018). “Great southern lands: making space for teacher resilience in South Africa and Australia” in Resilience in Education: Concepts, Contexts and Connections. eds. Wosnitza M. Peixoto F. Beltman S. Mansfield C. F. (Cham: Springer), 53–71.

[ref47] Masten A. Best K. Garmezy N. (1990). Resilience and development: contributions from the study of children who overcome adversity. Dev. Psychopathol. 2, 425–444. doi: 10.1017/S0954579400005812

[ref48] Mercer S. Kostoulas A. (2018). “Introduction to language teacher psychology,” in Language Teacher Psychology. eds. Mercer S. Kostoulas A. (Bristol, Blue Ridge Summit: Multilingual Matters), 1–17.

[ref49] Miles M. B. Huberman A. M. Saldaña J. (2014). Qualitative Data Analysis: An Expanded Sourcebook (3rd Edn.). Thousand Oaks, California: Sage Publications, Inc.

[ref50] Murray N. Liddicoat A. J. Zhen G. Mosavian P. (2020). Constraints on innovation in English language teaching in hinterland regions of China. Lang. Teach. Res. 1–22. doi: 10.1177/1362168820979855

[ref51] Pretsch J. Flunger B. Schmitt M. (2012). Resilience predicts well-being in teachers, but not in non-teaching employees. Soc. Psychol. Educ. 15, 321–336. doi: 10.1007/s11218-012-9180-8

[ref52] Richards K. A. R. Levesque-Bristol C. Templin T. J. Graber K. C. (2016). The impact of resilience on role stressors and burnout in elementary and secondary teachers. Soc. Psychol. Educ. 19, 511–536. doi: 10.1007/s11218-016-9346-x

[ref53] Rutter M. (1987). Psychosocial resilience and protective mechanisms. Am. J. Orthop. 57, 316–331. doi: 10.1111/j.1939-0025.1987.tb03541.x 3303954

[ref54] Tait M. (2008). Resilience as a contributor to novice teacher success, commitment, and retention. Teach. Educ. Q. 35, 57–75.

[ref55] Talbot K. Mercer S. (2018). Exploring university ESL/EFL teachers’ emotional well-being and emotional regulation in the United States, Japan and Austria. Chin. J. Appl. Linguist. 41, 410–432. doi: 10.1515/cjal-2018-0031

[ref400] Theron L. C. (2018). “Teacher championship of resilience: Lessons from the pathways to resilience study, South Africa,” in Resilience in Education: Concepts, Contexts and Connections. eds. Wosnitza M. Peixoto F. Beltman S. Mansfield C. F. (Cham: Springer), 203–220.

[ref56] Ushioda E. (2020). Language Learning Motivation: An Ethical Agenda for Research. Oxford: Oxford University Press

[ref57] Wang Y. Derakhshan A. Rahimpour H. (2022). Developing resilience among Chinese and Iranian EFL teachers: a multi-dimensional cross-cultural study. J. Multiling. Multicult. Dev., 1–18. doi: 10.1080/01434632.2022.2042540

[ref58] Wen Q. Zhang H. (2020). “China going global: challenges and responses in English as a foreign language teaching and teacher education,” in English Language Teaching and Teacher Education in East Asia: A Global Challenges and Local Responses. ed. Tsui A. B. M. (New York: Cambridge University Press), 1113–1134.

[ref59] Wosnitza M. Peixoto F. (2018). “Resilience in education: emerging trends in recent research” in Resilience in Education: Concepts, Contexts and Connections. eds. Wosnitza M. Peixoto F. Beltman S. Mansfield C. F. (Cham: Springer), 335–340.

[ref60] Yu X. Zhang J. (2007). Factor analysis and psychometric evaluation of the Connor-Davidson resilience scale (CD-RISC) with Chinese people. Soc. Behav. Personal. 35, 19–30. doi: 10.2224/sbp.2007.35.1.19

[ref61] Zhang M. (2021). EFL/ESL teacher’s resilience, academic buoyancy, care, and their impact on students’ engagement: a theoretical review. Front. Psychol. 12:731859. doi: 10.3389/fpsyg.2021.731859, PMID: 34484087 PMC8416253

